# Quasi one-dimensional band dispersion and surface metallization in long-range ordered polymeric wires

**DOI:** 10.1038/ncomms10235

**Published:** 2016-01-04

**Authors:** Guillaume Vasseur, Yannick Fagot-Revurat, Muriel Sicot, Bertrand Kierren, Luc Moreau, Daniel Malterre, Luis Cardenas, Gianluca Galeotti, Josh Lipton-Duffin, Federico Rosei, Marco Di Giovannantonio, Giorgio Contini, Patrick Le Fèvre, François Bertran, Liangbo Liang, Vincent Meunier, Dmitrii F. Perepichka

**Affiliations:** 1 Institut Jean Lamour, UMR 7198, Université de Lorraine/CNRS, BP 70239, F-54506 Vandoeuvre-les-Nancy, France; 2 Centre Énergie, Matériaux et Télécommunications, Institut National de la Recherche Scientifique, 1650 Boulevard Lionel-Boulet, Varennes, Quebec, Canada J3X 1S2; 3 IRCELYON, Institut de Recherches sur la Catalyse et l'Environnement de Lyon, Villeurbanne 69626, France; 4 Institute for Future Environments, Queensland University of Technology (QUT), 2 George Street, Brisbane, Queensland 4001, Australia; 5 Institute for Fundamental and Frontier Science, University of Electronic Science and Technology of China, Chengdu 610054, China; 6 Instituto di Struttura della Materia, CNR, Via Fosso del Cavaliere 100, 00133 Roma, Italy; 7 Physics Department, University of Rome ‘Tor Vergata', Via della Ricerca Scientifica 1, I-00133 Roma, Italy; 8 Synchrotron SOLEIL, L'Orme des Merisiers, Saint-Aubin, BP 48, F-91192 Gif sur Yvette, France; 9 Department of Physics, Applied Physics, and Astronomy, Rensselaer Polytechnic Institute, Troy, NY 12180, USA; 10 Center for Nanophase Materials Sciences, Oak Ridge National Laboratory, Oak Ridge, Tennessee 37831, USA; 11 Department of Chemistry, McGill University, 801 Sherbrooke Street West, Montreal, Quebec, Canada H3A 0B8

## Abstract

On-surface covalent self-assembly of organic molecules is a very promising bottom–up approach for producing atomically controlled nanostructures. Due to their highly tuneable properties, these structures may be used as building blocks in electronic carbon-based molecular devices. Following this idea, here we report on the electronic structure of an ordered array of poly(para-phenylene) nanowires produced by surface-catalysed dehalogenative reaction. By scanning tunnelling spectroscopy we follow the quantization of unoccupied molecular states as a function of oligomer length, with Fermi level crossing observed for long chains. Angle-resolved photoelectron spectroscopy reveals a quasi-1D valence band as well as a direct gap of 1.15 eV, as the conduction band is partially filled through adsorption on the surface. Tight-binding modelling and *ab initio* density functional theory calculations lead to a full description of the band structure, including the gap size and charge transfer mechanisms, highlighting a strong substrate–molecule interaction that drives the system into a metallic behaviour.

## Abstract (web summary)

Polymerization on surfaces is an emerging approach for producing graphene nanoribbons with a tunable bandgap, a promising material for carbon-based electronics. Here, Vasseur *et al.* show quasi-one-dimensional band structure of a model semiconducting polymer synthesized directly on a supporting surface.

## Graphical abstract

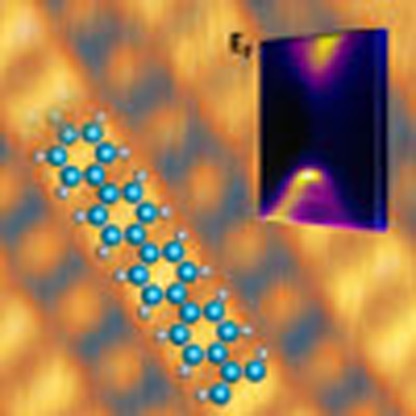

A major challenge in modern surface science is to create ordered arrays of covalently linked organic nanostructures. By doping molecular electronic bands into highly conductive states, these structures may be promising for use as elementary building blocks in electronic carbon-based molecular devices such as organic field-effect transistors^1^, light-emitting diodes^2^,^3^, photovoltaics^4^ and sensors^5^. Despite its exceptional physical properties, graphene's lack of a bandgap severely limits its potential for creating such devices. Engineering the gap in graphene by using nanostructuring, for example, creating graphene nanoribbons (GNRs) of narrow width, has been proposed as a feasible route towards carbon-based electronics. Thus, the GNRs' bandgap can be tuned by altering their lateral size or by modifying their edge termination (armchair versus zigzag)^6^,^7^,^8^. An emerging bottom–up approach for producing such carbon nanostructures, exploits covalent linking (polymerization) of precursor molecules on metal surfaces^9^,^10^,^11^,^12^,^13^,^14^,^15^,^16^,^17^,^18^,^19^,^20^. In these materials, functional properties, including the geometry and the bandgap, can be tailored by means of a judicious choice of monomer and supporting surfaces^21^,^22^,^23^,^24^. The on-surface polymerization is typically demonstrated by measuring the periodicity of polymeric architectures using scanning tunnelling microscopy (STM)^25^. Evidence of *π*-conjugation was shown by combining X-ray photoelectron spectroscopy and near-edge X-ray absorption fine structure^26^,^27^,^28^,^29^ (NEXAFS). Bandgaps can be deduced by scanning tunnelling spectroscopy (STS) and/or angle-resolved photoelectron spectroscopy (ARPES) and supported by theoretical calculations^30^,^31^,^32^,^33^,^34^,^35^. However, a full-band dispersion in polymeric chains has not been reported to date, due to the difficulty in obtaining ordered phases at sufficiently long range.

In this work, we unambiguously establish the full-band structure of a surface-confined *π*-conjugated organic polymer, as well as the impact of the substrate on its electronic properties. A long-range ordered array of poly(para-phenylene) (PPP) chains was produced through the surface-catalysed dehalogenative polymerization of 1,4-dibromobenzene (dBB) on copper (110). The high structural quality of the molecular layer, combined with the large extent of the individual PPP oligomers permitted both local and surface-averaged studies. Energy-dependent standing wave patterns observed by STS in finite-size PPP oligomers allowed the determination of the *k*-resolved conduction band dispersion. The conduction band is observed to cross the Fermi level, conferring to the polymer a metallic character. Using ARPES, we measured the valence band structure along the chains spread over 6.7 eV. As the conduction band is partially occupied, a 1.15 eV bandgap was directly observed. A Hückel/tight-binding (TB) model provides understanding of both ARPES and STS measurements, allowing the estimation of both effective intra- and interchains resonance integrals and establishes the quasi-one-dimensional (1D) nature of the dispersion. First-principles density functional theory (DFT) calculations fully reproduce the band structure and point out a strong hybridization at the organic/metal interface, which is responsible for filling the polymer's unoccupied states.

## Results

### An ordered and commensurate polymeric phase on Cu(110)

**Figure 1:**
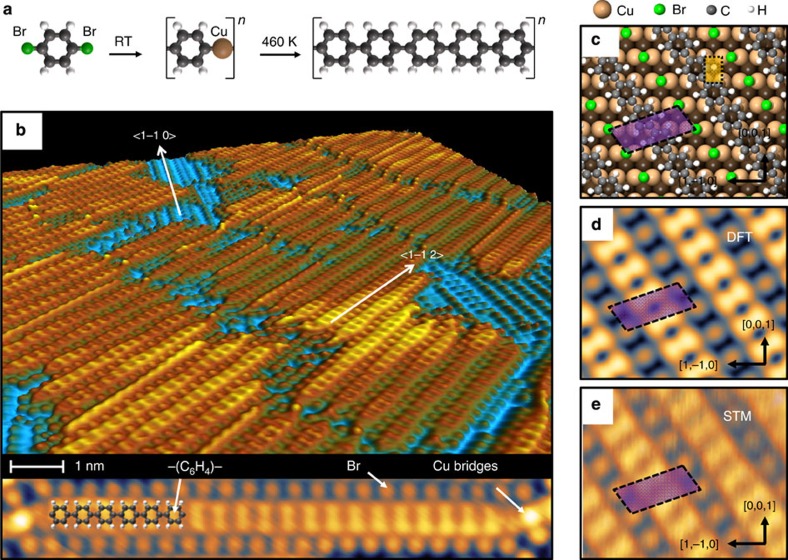
Growth of the poly(para-phenylene) chains on Cu(110). (**a**) Ullmann coupling reaction for 1,4-dibromobenzene on Cu(110); two additional Br atoms linked to the metallic surface (not shown here for clarity), are produced by the Ullmann reaction as experimentally observed. (**b**) 3D rendering STM image of the poly(para-phenylene) chains grown on Cu(110) for coverage close to 1 monolayer after annealing to 500 K (*I*=0.2 nA, *U*=0.1 V). Blue area contains additional Br atoms adsorbed on the Cu substrate. The bottom part shows the detailed structure of the PPP chains separated by bromine lines; (**c**) DFT-optimized geometry of the polymerized system. Atoms are represented as spheres according to the legend. The orange and violet areas are the Cu(110) and PPP/Cu(110) surface unit cells, respectively. Simulated (**d**) and experimental (**e**) STM images of the surface recorded at +0.2 eV.

A systematic investigation of the dBB/Cu(110) interface as a function of coverage and annealing temperature allows us to identify an unreported structural arrangement, which was used as a starting point for the formation of an ordered polymer phase via thermal activation. In previous work, Di Giovannantonio *et al.*^26^ demonstrated that the thermal treatment of vacuum-deposited dBB on Cu(110) leads to the formation of PPP chains. This process is understood to be an Ullmann coupling reaction, which is summarized in Fig. 1a: depositing the molecules at room temperature leads to an organometallic phase^26^,^36^, where Br–C bonds are replaced by C–Cu–C bridges. Subsequent annealing above 460 K promotes C–C bonding, resulting in the formation of coaligned *π*-conjugated PPP chains with a lattice parameter of 4.4 Å as measured by STM. In that case, the PPP chains were aligned along the <1–10> direction, for which the interatomic Cu distance of the surface is 2.55 Å. This leads to the incommensurability between the polymer and the substrate (Supplementary Fig. 3, Supplementary Note 2). However, it is possible to obtain an additional organometallic phase at high coverage, above 0.9 monolayer, which co-exists with the organometallic phase described above and fully dominates the surface at 1 monolayer. STM images and low-energy electron diffraction patterns characterising these two phases are provided and discussed in the Supplementary Figs 1 and 2 and Supplementary Note 1. When this high-coverage phase is annealed at 500 K, alternating rows of polymers and bromine atoms are formed, as shown in the STM images presented in Fig. 1b. The measured internal periodicity of 4.4±0.2 Å agrees with the expected phenyl–phenyl spacing, which is a characteristic of PPP. The polymer chains produced from high coverage are packed into domains and aligned along the two <1–1–2> and <1–12> substrate directions, a detail that complicates measurement by surface-averaging techniques such as photoelectron spectroscopy. While a single-domain orientation dominates in Fig. 1b, both orientations are observed in longer range STM images, and cover the surface with equal probability (Supplementary Fig. 4). The substrate periodicity along <1–12> is 4.43 Å, which matches with the lattice parameter of the PPP. Thus, the polymer is here fully commensurate with the surface and may be grown to long size with little or no strain arising from lattice mismatch (Supplementary Note 3).

The results of DFT optimization of the surface structure are presented in Fig. 1c (see Methods section for details)^37^,^38^,^39^. The GGA–PBE formulation was used to approximate the exchange-correlation functional potential. The van der Waals (vdW) interactions were described using the vdW density functional (vdW-DF) together with the optB86b exchange functional^40^. This approach yields a very good description of the vdW forces between molecules and surfaces^41^. The PPP polymer (C atoms grey) is adsorbed in a flat geometry, 2.2 Å above the copper surface. Such a short distance (cf. 3.35 Å for inter-planar spacing in graphite) points to a significant hybridization of the electronic levels of the polymer with the copper interface states. Bromine atoms (green spheres) are adsorbed on the short bridge (SB) site of the underlying Cu lattice (orange spheres). The centre of each phenyl ring overlaps the hollow (H) site, which is the preferred site for benzene adsorption on Cu(110)^42^. An STM image calculated using the Tersoff–Hamann approximation^43^ at a bias voltage of 200 mV is an excellent match to the experimental data as presented in Fig. 1d,e.

### Local spectroscopy and tight-binding modelling

**Figure 2:**
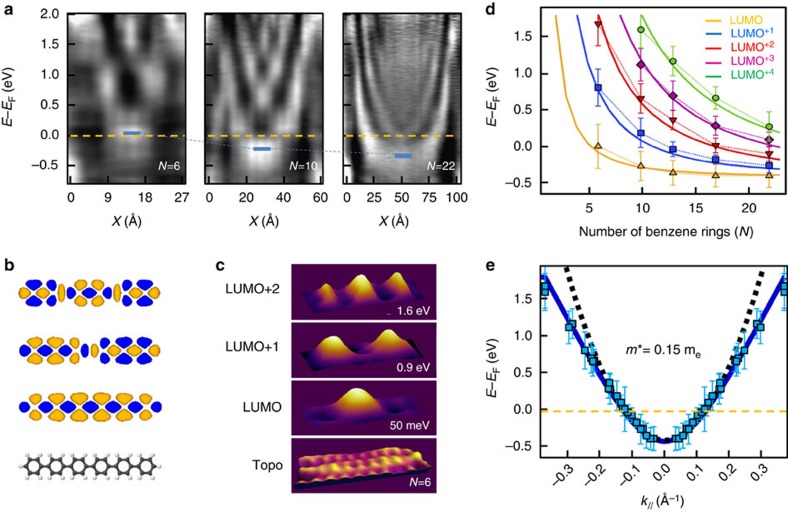
Conduction band structure deduced by scanning tunnelling spectroscopy. (**a**) Experimental d*I*/d*V* measurements recorded along a polymer for three lengths (*N* correspond to the number of phenyl rings). The vertical axis is energy, the horizontal axis is position along the polymer. The blue marks show the bottom of the bands. (**b**) Schematic representation of the free sexiphenyl molecule and its LUMO, LUMO+1 and LUMO+2 states calculated using a tight-binding model built from Hückel orbitals. (**c**) Experimental topography (bottom) and STS conductance maps for the *N*=6 case. (**d**) Experimental (dots) and theoretical (solid lines, extracted from Hückel's model) evolution of the energy of the LUMO states as function of the length of the polymeric chains. Error bars are given by the s.d. extracted from Gaussian fits (Supplementary Fig. 4). (**e**) Experimental and theoretical dispersion of the LUMO conduction band. Experimental points (blue) are extracted from data shown in **d**, using the relation *k*_//_=2*πp*/Na (*p*=1,2,…). The theoretical band (blue solid line), is obtained from a tight-binding model using the same resonance integral as the Hückel model. The black dashed curve represents a parabolic dispersion with m*=0.15±0.02 *m*_e_. In **d** and **e**, absolute positions of calculated bands have been shifted by 1.9 eV towards occupied states to match the experimental results.

Using STS, confinement of unoccupied molecular states has been observed previously in individual polythiophene chains^44^, and more recently, in 7-AGNRs^35^ and GNR heterojunctions^45^. We recorded differential conductivity maps as a function of chain length, to build the *k*-resolved band structure associated with the conduction band of the infinite polymer. STS measurements obtained on PPP oligomers with *N*=6 (26 Å), *N*=10 (44 Å) and *N*=22 (97 Å) phenyl rings are presented in Fig. 2a. As expected, the local density of states (LDOS) is spatially modulated along the chains, leading to standing wave patterns which depend strongly both on the bias voltage and the oligomer length. For *N*=6, the lowest unoccupied molecular orbital (LUMO; indicated by a blue segment in Fig. 2a) is slightly above the Fermi level. As the length of the chain is increased, the LUMO progressively moves below the Fermi level, reaching −0.4 eV for the *N*=22 polymer.

A simple TB model (see Methods section for details) reproduces our results. The long-range modulations observed in the LUMO, LUMO+1 and LUMO+2 orbitals are in agreement with the local conductance maps recorded for the first three states in the polymer chains, as shown in Fig. 2b,c for the *N*=6 case. Using a single C–C resonance integral *β*=−3.6 eV, an excellent fit to experimental binding energies (Supplementary Fig. 4 and Supplementary Note 3) as a function of oligomer length is observed as presented in Fig. 2d. However, the absolute position of these levels is not well described by the model and has to be manually shifted by 1.9 eV towards occupied states. Defining **k**_//_=2*pπ*/Na (*p*<*N*, *a*=4.4 Å) as the wave vector associated to the 1D-confined states allows us to build the conduction band dispersion for the infinite polymer, as shown in Fig. 2e. A parabolic fit (black dashed line) to the bottom of the conduction band implies an effective mass of 0.15±0.02 *m*_e_ for charge carriers in the vicinity of the Fermi level. This value is in agreement with those obtained for AGNRs^30^. As the band crosses the Fermi level at **k**_F_=±0.12 Å^−1^, the PPP polymers are metallic and thus represent an array of conducting nanowires. None of the occupied states were resolved by STS, and thus we were unable to extract the bandgap of the PPP.

### Band structure from angle-resolved photoelectron spectroscopy

**Figure 3:**
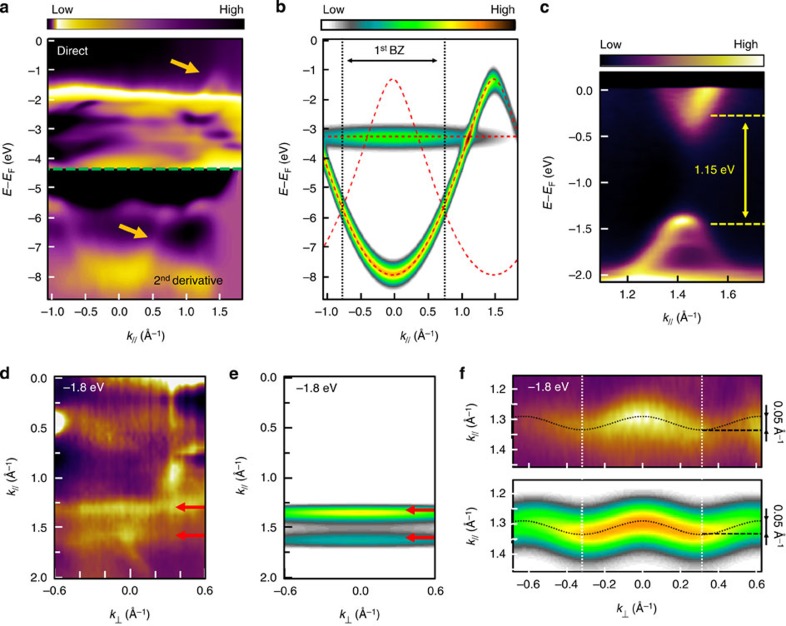
Angle-resolved photoelectron spectroscopy measurements. (**a**) ARPES intensity map measured along the <1–12> direction, parallel to the polymers chains for 1ML-PPP/Cu(110). The bottom part is displayed in second derivative. Arrows indicate bands due to the presence of polymers. (**b**) Tight-binding modelling of the band structure of the *N*=20 PPP polymer using *β*=−3.5 eV. (**c**) High-resolution ARPES intensity map recorded close to the Fermi level showing the HOMO–LUMO bandgap. Measurements (**d**) and tight-binding modelling (**e**) of the ARPES constant energy map at −1.8 eV. (**f**) Zoom on the measured (top) and calculated (bottom) ARPES constant energy maps. The transverse resonance integral used in the calculations is *β*′=−0.15 eV.

ARPES has previously been used to reveal quantum well states in molecules^46^,^47^ or to reconstruct the discrete electronic orbitals of self-assembled individual molecules at surfaces^48^,^49^. In addition, evidence of changes in the density of states of polymers, especially as a function of doping, have been observed by ultraviolet photoelectron spectroscopy (UPS)^50^. Nevertheless, direct evidence of a *k*-resolved band structure associated with the C–C covalent bonding and a long-range delocalization of charge carriers have been reported to date only on graphene materials^31^. Here ARPES intensity maps were measured along the <1–12> axis, parallel to the chain direction of one domain (therefore probing roughly half of the monolayer). The incident photons were *p*-polarized, with an energy of *hν*=35 eV. The spectral intensity from the second domain, which is rotated by 70.52° with respect to the first, does not contribute to the ARPES signal because its *k*-points are not accessible in this experimental geometry, except at the Γ point. Comparison of surfaces both with and without polymers permits the identification of two dispersive bands, on both sides of the 3*d* states of the substrate, labelled in Fig. 3a using yellow arrows. The bottom of the first band is located at the Γ point at *E*−*E*_F_=−8.1 eV, whereas the top of the second one reaches a maximum at *E*−*E*_F_=−1.4 eV at *k*_//_=1.4 Å^−1^. The absence of these bands in the clean Cu(110) spectra confirms their molecular origin (Supplementary Fig. 5). In addition, a strong decrease of the ARPES signal is observed when the photon polarization vector is changed from *p*-polarization (having an out of surface plane component of the polarization vector parallel to *π*-orbital axis) to *s*-polarization (having only in surface plane component of the polarization vector) consistent with the *π*-character of the molecular orbitals and the flat-lying geometry of the phenyl rings (Supplementary Note 4, Supplementary Fig. 5). Due to the high intensity of Cu 3*d* bands it is not possible to resolve the molecular dispersion in the energy range *E*−*E*_F_ between −2 and −5 eV.

Calculation of band structure and ARPES spectral intensity using the Hückel (tight-binding) models provides an understanding of the observed dispersions. The valence band structure of the infinite free polymer, calculated in the TB model using a resonance integral of *β*=−3.5 eV, is presented in Fig. 3b (red dashed lines). Here the molecular band was rigidly shifted by 0.1 eV to match the data. Three bands are easily identified, two of them dispersing in opposite phase and crossing at the edge of the first Brillouin zone (BZ) of the system, that is, for *k*_//_=±*π*/*d*=0.71 Å^−1^, consistent with the PPP periodicity of *d*=4.4 Å. The theoretical ARPES intensity (colour scale on Fig. 3b) was deduced from the Fourier-transform of each molecular orbitals calculated for a *N*=20 polymer, according to the Fermi's golden rule as proposed in the cases of sexyphenyl on the Cu(110) surface^41^ and PTCDA on the Ag(110) surface^42^ (Supplementary Figs 6 and 7, Supplementary Note 5). It appears that, as usually observed in ARPES, the spectral intensity does not follow the periodicity of the reciprocal lattice, due to cross-sectional effects in the photoelectron emission^51^,^52^. In particular, the ARPES intensity of the top band at the gamma point of the first BZ is equal to zero, and thus no signature of this band was observed in normal emission. This phenomenon leads to an apparent band periodicity of 2.84 Å^−1^, which does not appear to match the BZ size (1.42 Å^−1^). Therefore, knowledge of the orbital topology and a rudimentary model of the electronic structure are necessary to explain the appearance of experimental dispersion curves.

High-resolution measurements taken in the second BZ close to the top of the occupied molecular band show that the molecular spectral intensity disperses up to a binding energy of −1.4 eV at *k*_//_=1.42 Å^−1^, while a small portion of a higher energy band dips below the Fermi level. A direct bandgap of 1.15 eV is found between the minimum of this band and the top of the valence band described above. This result is in agreement with the location of the conduction band as revealed by our STS measurements on long PPP polymers, confirming the metallic character of the polymer.

In addition, a constant energy map taken at *E*−*E*_F_=−1.8 eV, close to the molecular band maximum, is shown in Fig. 3d (Supplementary Fig. 8). Two almost completely linear contributions are clearly identified at well-defined *k*_//_ values. However, a detailed investigation shows that the perpendicular dispersion is not precisely zero, as a periodicity of *k*_⊥_=0.6 Å^−1^=2*π*/*b* (where *b*=10.4 Å, the distance between adjacent PPP chains) is apparent in Fig. 3f. A TB model incorporating an effective interchain hopping integral *β*′=−0.15 eV permits the quantification of this small dispersion (Supplementary Note 6). A weak transverse dispersion such as this may correspond to indirect hopping mediated by the substrate and/or by Br atoms^53^. We thus extract an experimental ratio *β*′/*β*≈0.04, that allows us to conclude on the quasi-1D nature of the molecular bands.

### DFT calculations

**Figure 4:**
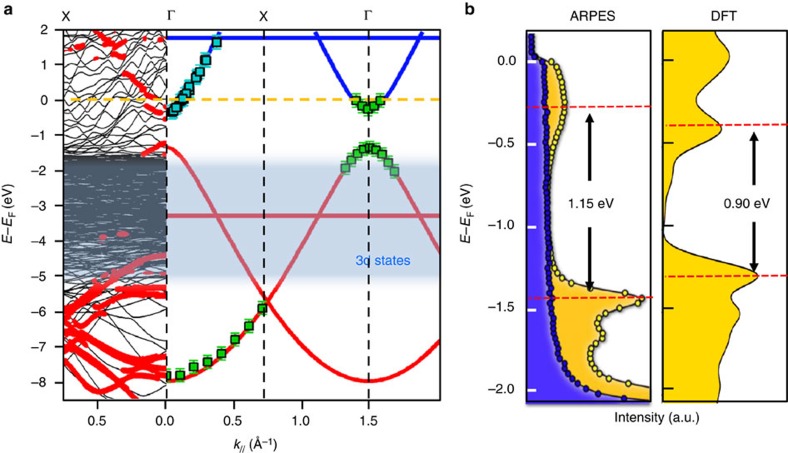
Band-structure of PPP polymers grown on Cu(110). (**a**) DFT band structure (black lines, red circles correspond to the projection on the molecular layer), tight-binding modelling (red and blue solid lines), ARPES (green squares) and STS (blue squares) experimental dispersion curves. The absolute positions of the valence and conduction bands in the tight-binding model have been independently shifted towards the occupied states by 0.1 and 1.9 eV, respectively. (**b**) *k*-integrated photoemission DOS on the Cu(110) substrate (left panel, blue), on PPP/Cu(110) (left panel, yellow) and corresponding DFT DOS (right panel, yellow).

In Fig. 4a (right part), we show that our local STS measurements (blue squares) for different chain lengths are in remarkable agreement with the *k*-resolved conduction band structure obtained from ARPES (green squares). Red and blue solid lines present the band structure calculated in the TB model using a single C–C resonance integral *β*=−3.5 eV in agreement with the expected value for graphene materials^54^. However, the valence and conduction bands are independently shifted in energy, respectively, by 0.1 and 1.9 eV toward occupied states, since our TB model leads to a bandgap of 2.9 eV. So far, the precise value of the gap (1.15 eV) and the partial filling of the unoccupied molecular states cannot be understood in the framework of this simple yet powerful method. DFT computation of the electronic band structure for the model appearing in Fig. 1c is depicted at the left Fig. 4a. The bands originating from the occupied and unoccupied molecular states can be distinguished from those originating from the copper substrate by plotting the projection of the band structure onto the top layer of the system, which contains only H, Br and C atoms. The result of this projection is shown as red circles in Fig. 4a. The conduction band as well as several valence bands can be clearly identified. However, the projected band structure contains signatures of hybridization with the substrate, which are not captured in the TB model. A comparison between the measured and calculated electronic density of states (DOS) is shown in Fig. 4b. The DOS of pristine Cu(110) (filled blue line) is flat between 0 and −2 eV, and originates from copper *s*–*p* bands. The additional spectral intensity arising from the PPP overlayer appears clearly as peaks corresponding to the top and bottom of the valence and conduction molecular bands, respectively. The separation between these peaks corresponds to a bandgap of 1.15 eV. The calculated gap is ∼0.90 eV, which is slightly smaller than the experimental value. This is considered as a common drawback of DFT calculations, which neglect correlation effects. Overall, both the size of the gap and the minimum of the conduction band below the Fermi level are captured by these simulations, in agreement with the finding of metallic character in the PPP nanowires.

## Discussion

The gap for freestanding PPP is expected to be 2.9 eV on the basis of the TB approach^55^. Assuming the PPP polymer as being a 3*p*-type AGNR with *p*=1 and *w*=2.4 Å (Supplementary Fig. 9, Supplementary Note 7), we expect a bandgap of 2.45 eV from DFT calculations corrected at 4.1 eV including GW corrections^6^. We have also carried out DFT calculations in the gas phase using B3LYP/6–31g(*d*) that yield a bandgap of 3.05 eV (Supplementary Note 8). In another study, STS measurements were performed on isolated PPP chains grown on Cu(111) using a different precursor^32^. The bottom of the conduction band was found to be 0.9 eV above the Fermi level for long chains, in the unoccupied electronic states, that is, shifted by 1.3 eV compared with our measurements. Thus, understanding the interaction between the polymer and the substrate appears to be of major importance so as to explain both the bandgap closing and the filling of unoccupied molecular states, as emphasized very recently^34^.

**Figure 5:**
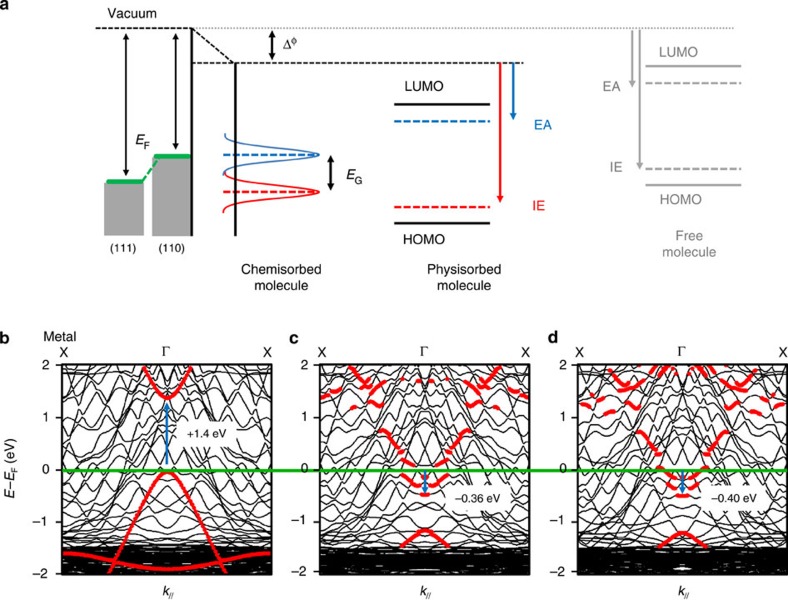
Impact of the substrate and bromine atoms on electronic properties. (**a**) Schematic representation of energy level alignments and bandgap modifications in organic/metal interfaces. DFT band-structure calculated enlarging the PPP-Cu(110) surface distance to 8 Å instead of 2.2 Å for the optimized structure (**b**), keeping the surface distance to 2.2 Å with (**c**) and without (**d**) Br atoms at surface. Red circles in the band-structure correspond to the projection on the first (molecular) layer with the same arbitrary threshold.

Figure 5a dissects the essential mechanisms that should be taken into account in the energy level alignment and gap modifications in organic semiconductor/metal interfaces. Considering that the PPP polymer is adsorbed onto a surface, the resulting position of the molecular levels depends on the substrate work function as indicated on the left side of Fig. 5a. The Cu(111) work function (4.94 eV) is larger than that of Cu(110) (4.48 eV). Consequently molecular levels are shifted to lower binding energies on the Cu(110). This effect may be enhanced by an additional reduction of the work function Δ*ϕ*_s_ due to the compression of the electron cloud inside the metal substrate induced by the proximity of the molecular layer^56^,^57^,^58^. In our case, the short distance of 2.2 Å between the polymer and the surface may explain the large shift observed in the present work as compared with the report on Cu(111)^32^. However, it has also been shown that certain physisorbed molecules experience Fermi level pinning: in this case, charge transfer from the substrate to the LUMO creates a new surface dipole, which increases the work function until the LUMO lies with the Fermi energy, inhibiting its occupation^59^,^60^. For chemisorbed molecular materials, substantial molecule/substrate hybridization leads to a direct charge transfer, which favours LUMO occupation. This mechanism has been recently identified as being responsible for the metallization of pentacenequinone and pentacenetetrone molecules adsorbed on coinage metal surfaces^34^. In addition, several works have also reported a strong reduction of the HOMO–LUMO gap induced by the proximity of molecules with a metallic substrate, due to charge screening effects^59^,^60^. To test the impact of the substrate on the electronic properties, the band structure was recalculated in a geometry where the separation between the PPP and the substrate is increased from 2.2 to 8 Å (Fig. 5b). It is clear that: (i) the valence and conduction molecular bands are better defined, having lost the signatures of hybridization with the substrate; (ii) the full molecular band structure is shifted upwards, with the top of the valence band grazing the Fermi level; (iii) the gap has increased to ∼1.4 eV but still remains much lower than expected from gas phase calculations^6^. In general the local density approximation and generalized gradient approximation (GGA) underestimate band gaps of GNRs and organic polymers in gas phase as compared with GW calculations^6^. Nevertheless, the new position of the conduction band is above 1 eV as observed for PPP/Cu(111)^32^. Finally, we discuss the influence of the Br atoms on the electronic structure. To this end, we calculated the band structure both with (Fig. 5c) and without (Fig. 5d) Br atoms on the surface. When Br is not present at surface the conduction band minimum (CBM) of the polymer is only slightly lowered from −0.36 eV to −0.40 eV. This can be understood by the fact that Br atoms adsorbed on Cu(110) act as electron acceptors, depleting some electron density from the surface and thereby slightly impeding the charge transfer from the Cu to PPP. In addition, copper end atoms terminating each PPP chain may have a higher contribution to the PPP doping due to their stronger C–Cu bonding. Nevertheless, such a doping should strongly depend on the PPP length. Hence, from our STS study showing no evidence of modification of the LUMO filling as function of the chain lengths we conclude that this process is negligible. We therefore conclude that hybridization between molecular *π*-states and copper states is the essential mechanism that permits partial filling of the LUMO, the lowering of the bandgap and results in metallic behaviour for the PPP nanowires. This effect is enhanced by the fact that PPP polymers are aligned in such a way as to be commensurate with the Cu(110) substrate, which maximizes here the molecule/surface interaction in comparison to the PPP/Cu(111) interface.

In conclusion, We combine here molecular self-assembly and covalent bonding processes to synthesize long-range ordered arrays of PPP and characterized their full *π*-band structure. We demonstrate the quasi-1D metallic character of these chains grown in a commensurable way on the Cu(110) surface. A simple Hückel TB model provides a good understanding of both ARPES and STS measurements and allows estimating effective inter- and intrachains resonance integrals. In addition, DFT calculations evidence the strong hybridization existing between the polymer and the substrate, which explains both bandgap reduction and metallization of the chains. Despite the substantial hybridization, bands characterizing the PPP wires remain strongly quasi-1D leading to conducting polymeric nanowires. A future systematic investigation of the electronic properties as a function of the chemical nature of the substrate and its geometry, as well as the role of the halogen species could be useful to tune the bandgap and the metallicity. This work is of particular relevance in the context of covalent organic nanostructures design for potential applications in nanoelectronic devices. Thus, the complete band dispersion of the polymer may be considered as the spectroscopic fingerprint of the polymerization process of such molecules as dBB on surfaces where the Ullman reaction can be achieved like gold, silver or copper and also as a recent promising approach on semiconductors^61^,^62^.

## Methods

### Sample preparation

The experiments were carried out in ultrahigh vacuum at base pressures of 10^−10^ mbar or better. The Cu(110) crystal was prepared by repeated cycles of sputtering (Ar+,1 keV) and annealing (750 K). 1,4-dibromobenzene molecules (Sigma Aldrich, purity 98%) were sublimed onto the surface (held at room temperature), using a leak valve at partial pressures of 10^−8^ mbar. The PPP polymer presented here was obtained by reaching the saturation coverage of the surface. Under these conditions, a one monolayer organic layer was accumulated after *t*=9 min of deposition corresponding to 7 Langmuir. After deposition, the sample was post-annealed between 450 and 550 K for 5 min, to fully polymerize the system^26^.

### STM/STS and ARPES measurements

STM and STS experiments were carried out using an Omicron LT-STM at a temperature of 5 K. STM images were recorded at constant tunnelling current (0.2 nA) and constant bias voltage (applied to the sample). The d*I*/d*V* spectra were recorded in the open feedback loop mode (*V*_stab_=2 V) using the lock-in technique (peak to peak modulated voltage *V*_pp_=30 mV, *f*=1,100 Hz). Spectra were normalized by subtracting a background corresponding to the clean surface. ARPES experiments were performed at the CASSIOPEE beamline (synchrotron SOLEIL). The polymerization process and the orientation of the polymers were carefully analysed using the characteristic low-energy electron diffraction and X-ray photoelectron spectroscopy signatures (respectively, shown in Supplementary Figs 1 and 2, Supplementary Information and ref. 26). The data presented in Fig. 3 were measured at 30 K using a Scienta R-4000 high-resolution hemispherical electron analyser, with linearly polarized 35 eV photons (for the experimental geometry see Supplementary Fig. 5).

### Tight binding and first-principle band-structure calculations

The free-molecule orbitals and energy levels were obtained using a standard Hückel model. The hopping integral between two first neighbour carbon atoms was assumed to be constant and equal to *β*. The Coulomb integral *α* was also assumed to be the same for all atoms and was adjusted to rigidly shift the band structure. STS differential conductivity has been obtained for a *N*=6 oligomer by taking the square modulus of the TB-wave functions as a function of the quantized energies. Calculated ARPES intensity maps were obtained by taking the square modulus of the Fourier-transform of the calculated orbitals, according to the method presented in refs. 46, 48 The calculated ARPES spectral intensity shown in Fig. 3e was obtained by calculating orbitals of three chains of 20 phenyl rings with an interchain coupling constant *β*′ of −0.15 eV.

DFT calculations were performed using the plane-wave pseudopotential code VASP^37^,^38^,^39^. We used the GGA formulation to approximate the exchange-correlation functional potential proposed by Perdew, Burke and Enzerhof (PBE). The (vdW interactions were described using the vdW density functional (vdW-DF) together with the optB86b exchange functional (see more details about the vdW-DF procedure in the original work by Dion *et al.*^40^). This approach yields a very good description of the vdW interactions between molecules and surfaces^41^. The size of the unit cell was first obtained from the relaxation of a clean Cu(110) surface. The unit cell dimensions were 4.41519 × 12.7455 × 25.00 Å^3^ (*γ*=54.736 degrees). The slab contained 40 copper atoms in 8 layers. The five bottom layers were kept fixed during relaxation, at positions corresponding to the copper bulk. A vacuum region of at least 12 Å was maintained to avoid spurious interactions with periodic images. The electronic structure calculations were performed using a 6 × 2 × 1 Monkhorst–Pack grid to sample the BZ (this corresponds to six non-equivalent *k*-points). The pseudopotentials were expressed within the projector-augmented wave scheme with an energy cutoff of 500 eV and a smearing *σ*=0.05 eV. Once the candidate structures were relaxed within 0.001 eV Å^−1^, the corresponding (constant current) STM images were computed within the Tersoff–Hamann approximation^43^ where the current at a given tip position above the sample is expressed as the integral of the density of states between the Fermi energy and the applied potential. The band structure plots were prepared using the converged Kohn–Sham orbitals during a non-self-consistent run along the four high-symmetry directions of the first BZ corresponding to the unit cell described above (each direction was sampled using 60 points). The band structures presented here also include plots of the bands projected on the top layer of the unit cell. The local density of states plots were projected on individual top-layer atoms, as indicated in the figure legend. Additional DFT calculations were carried out in the gas phase using Gaussian 09 at the B3LYP/6–31G(d) level (Supplementary Note 8).

## Additional information

**How to cite this article:** Vasseur, G. *et al.* Quasi one-dimensional band dispersion and surface metallization in long-range ordered polymeric wires. *Nat. Commun.* 7:10235 doi: 10.1038/ncomms10235 (2016).

## Supplementary Material

Supplementary Information Supplementary Figures 1-9, Supplementary Notes 1-8 and Supplementary References
