# Supplementary material for: Quasi one-dimensional band dispersion and surface metallization in long-range ordered polymeric wires
Source: Nat Commun. 2016 Jan 4;7:10235. doi: 10.1038/ncomms10235 (PMC4725758; doi:10.1038/ncomms10235)
Supplement: Supplementary Information — Supplementary Figures 1-9, Supplementary Notes 1-8 and Supplementary References [file ncomms10235-s1.pdf]

Supplementary Figures

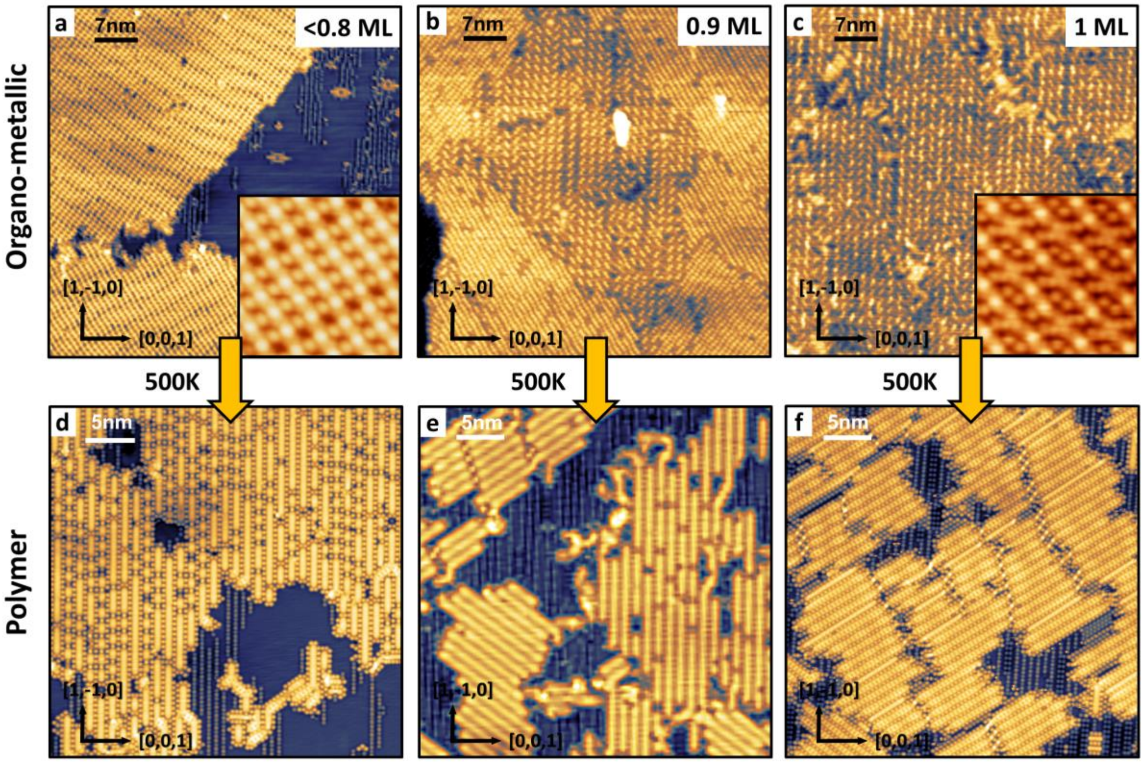

Supplementary Figure 1: Growth of the DBB on Cu(110) STM images of organometallic (a,b,c - 55 x 55 nm) and polymeric (d,e,f - 35 x 35 nm) molecular superstructures for different coverages : (a,d), lower than 0.8 ML, (b,e) around 0.9 ML, and (c,f) at saturation (1 ML). Insets in (a) and (e) show high resolution images of the two OM superstructures.

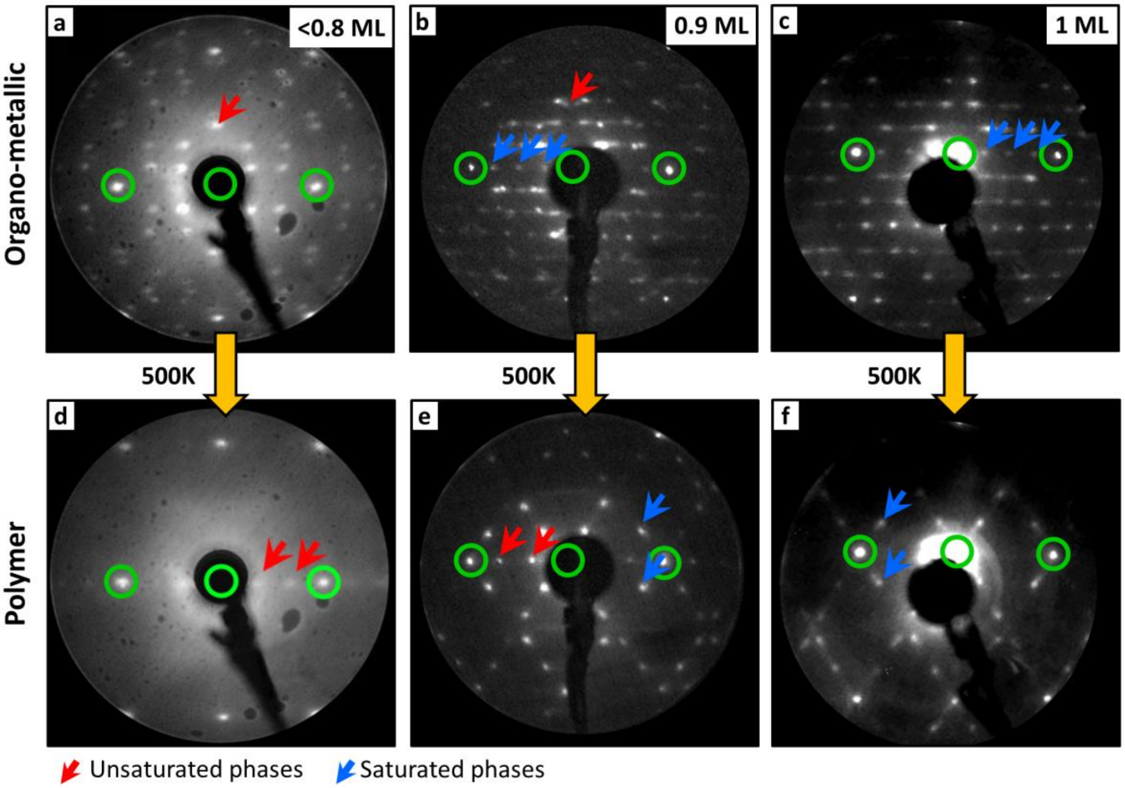

Supplementary Figure 2: LEED measurements for the different phases, recorded on the organometallic (a,b,c) and the polymer (d,e,f) phases, at different coverages : (a,d), below 0.8 ML, (b,e) around 0.9 ML, and (c,f) at saturation (1 ML).

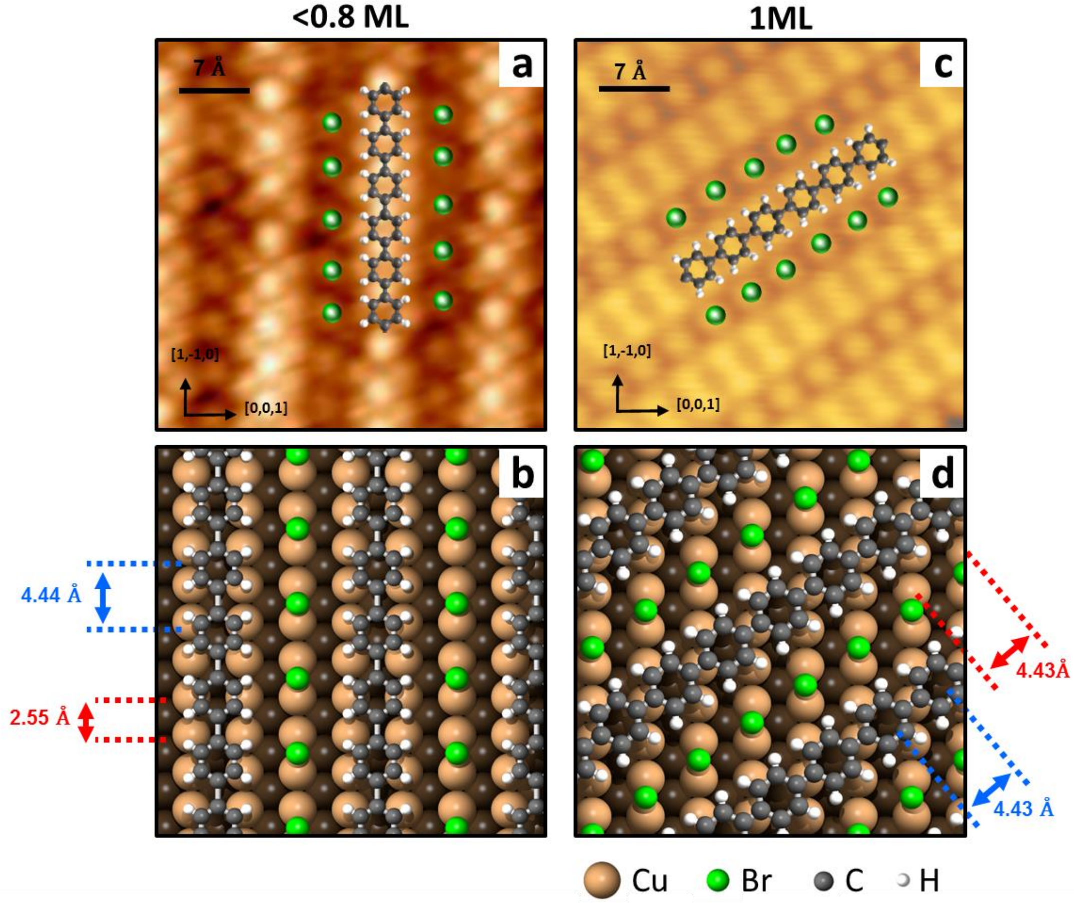

Supplementary Figure 3 : Structural difference between the polymer aligned in the [1,-1,0] and the [1,-1,2] directions. (a,c) High resolution STM images and (b,d) corresponding schematic view associated to the (a,b) [1,-1,0] and (c,d) [1,-1,2] aligned PPP polymers on Cu(110).

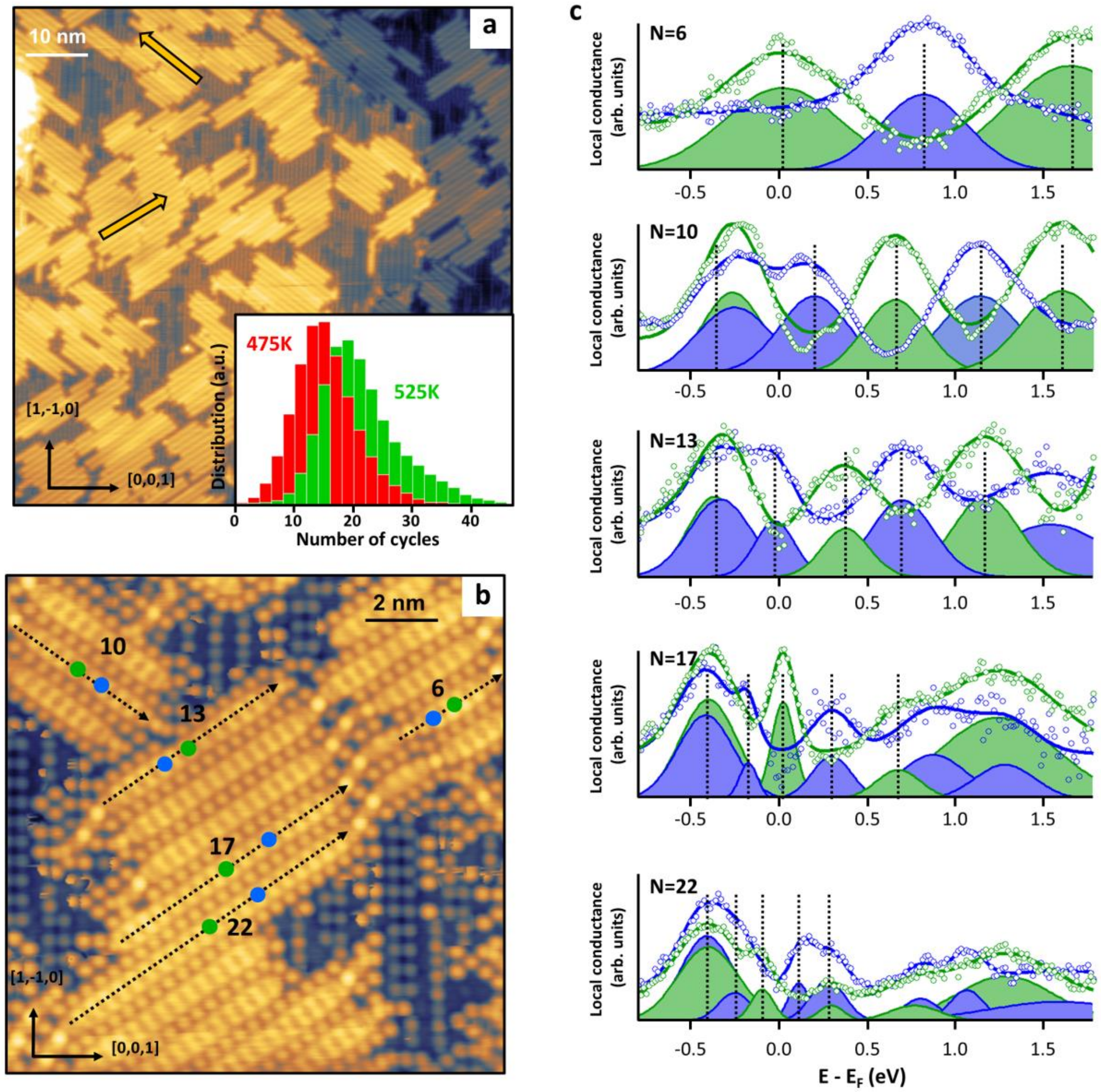

Supplementary Figure 4: Scanning tunneling spectroscopy measurements as function of the length of chains. (a) Large scale STM picture (80 x 80 nm) showing the molecular superstructures obtained after the annealing of a full monolayer of DBB on Cu(110) at 475 K during 5 min. (b) High resolution STM picture (14 x 14 nm) showing the 5 polymeric chains used for STS measurements presented in Figure 2. (c) experimental local conductance (points) measured as function of the chain's length N for different positions on the chain (L/2 and L/3, see colored points dispayed in (b)). Each spectrum is fitted (solid lines) removing a polynomial background and using several Gaussian contributions represented with solid areas. The positions of the five first LUMO states are highlighted using black dashed lines.

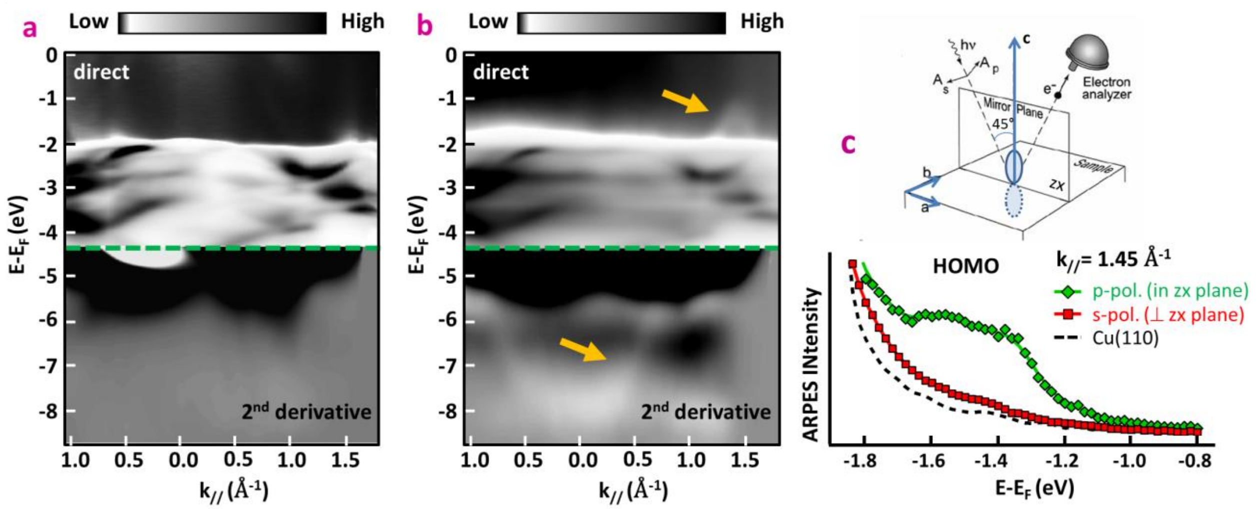

Supplementary Figure 5: **Comparison of ARPES measurements obtained before and after the polymer synthesis.** (a,b) ARPES intensity maps measured in the <1,-1,2> direction, parallel to the chains, for (a) the clean surface and (b) the annealed 1ML-BB on Cu(110). The bottom part is displayed in 2nd derivative view. (c) ARPES spectra measured at the top of the molecular dispersion ($k_{//}$=1.43Å^{-1}) for the clean and the polymer covered substrate, with a p-polarized (green points) and s-polarized (red points) light. The typical geometry of the ARPES experiment is shown on top.

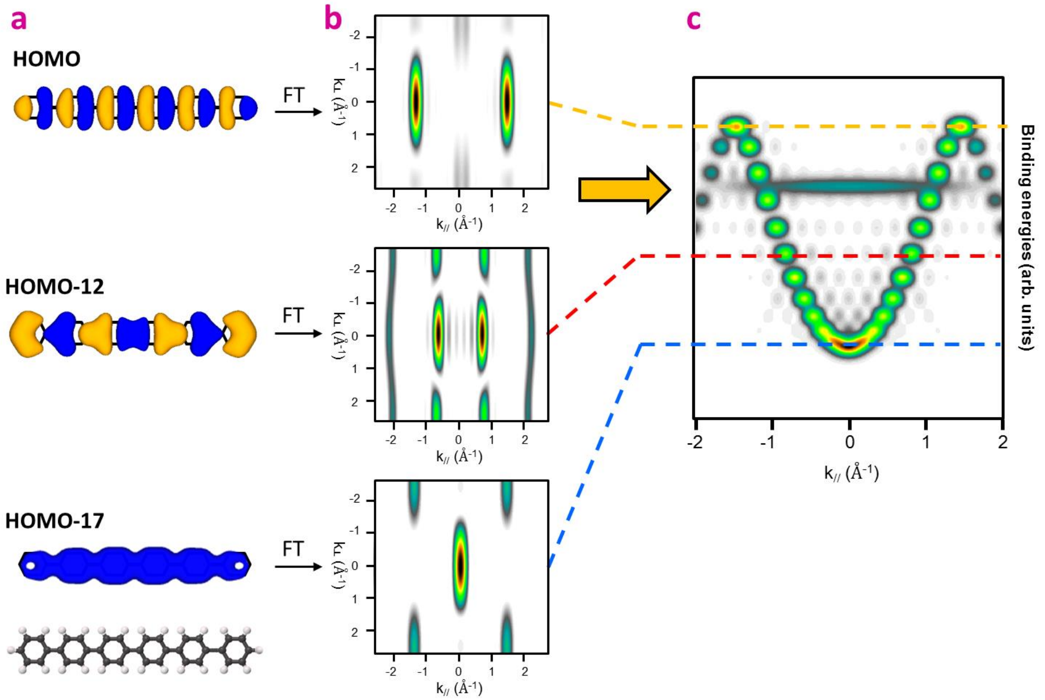

Supplementary Figure 6: Theoretical ARPES intensity calculated for the sexiphenyl. (a) 2D representation of HOMO, HOMO-12 and HOMO-17 of the sexiphenyl calculated using the Hückel method. (b) Corresponding calculated constant energy ARPES maps calculated according to reference 5. (c) Calculated E vs $k_{//}$ ARPES map calculated from the CEAMs .

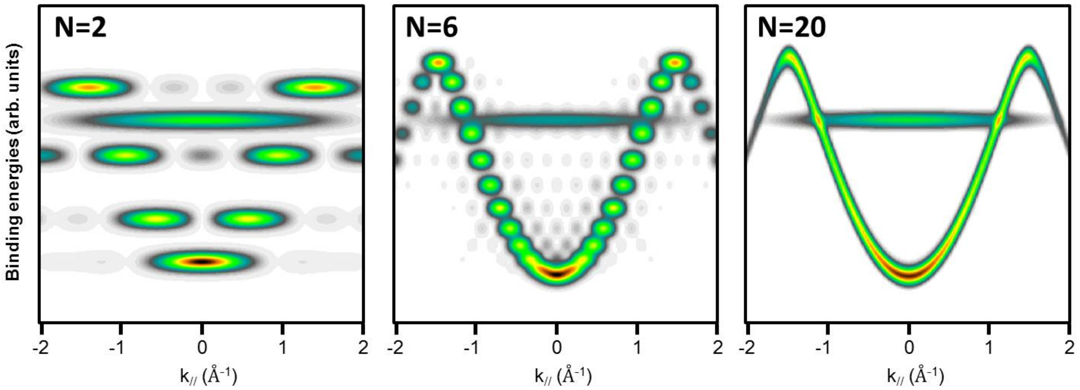

Supplementary Figure 7: Evolution of the ARPES intensity distribution as function of the oligomer length. Calculated E vs $k_{//}$ ARPES maps calculated for finite PPP oligomers with 2 (left), 6 (center) and 20 (right) phenyl rings.

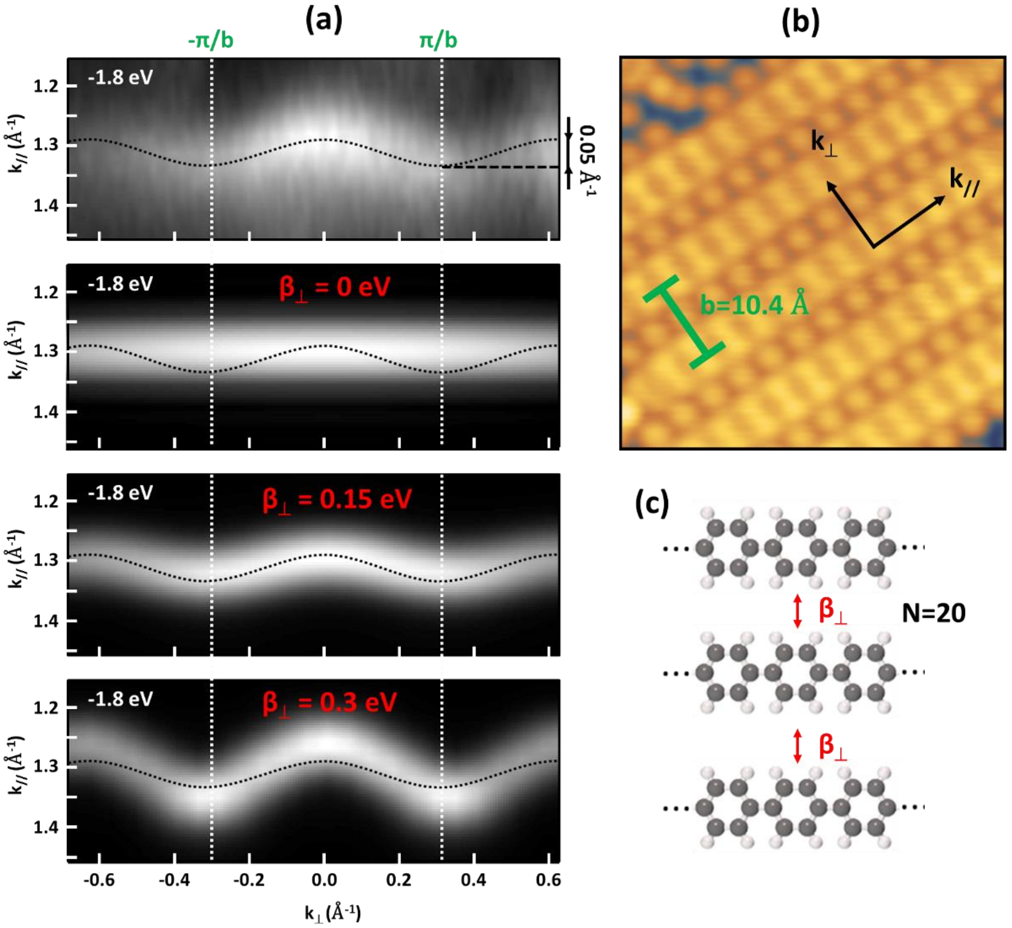

Supplementary Figure 8: Evolution of the ARPES intensity distribution as function of the inter-chain coupling. (a) Experimental (top part) and calculated constant energy ARPES maps (-1.8 eV) centered on the polymer contributions, showing the evolution of the modulation related to the perpendicular inter-chain coupling as function of β' (see text). (b) High resolution STM image showing four PPP chains separated by 10.4 Å in the perpendicular direction. (c) Schematic representation of the finite system used in Hückel calculations.

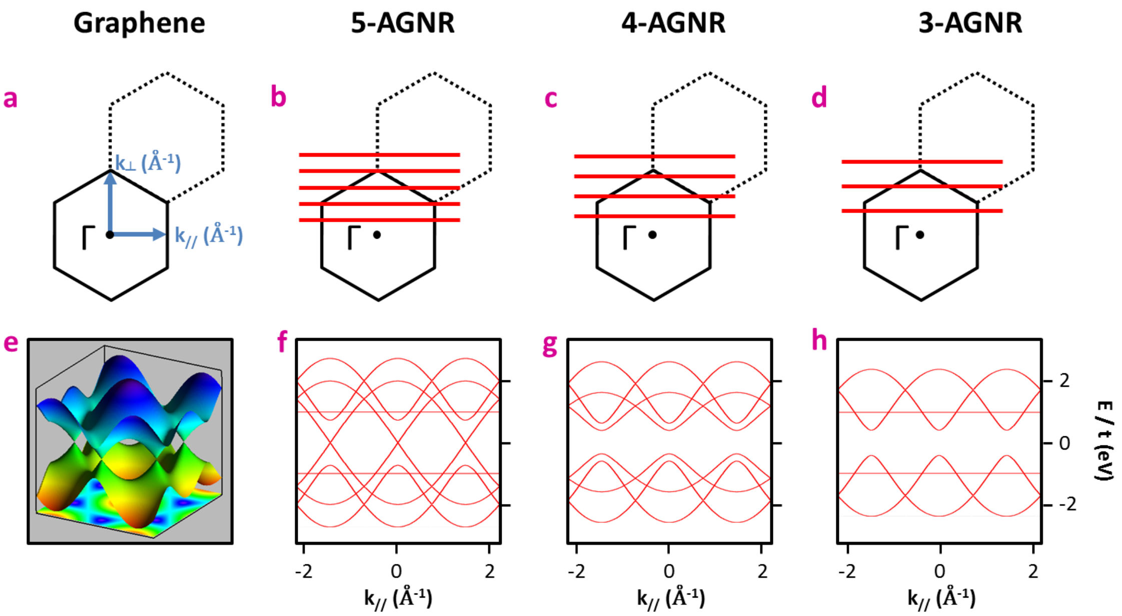

Supplementary Figure 9: Relation between the 2D graphene band structure and the ones of armchair graphene nanoribbons. (a-d) Schematic representation of the first (black solid line) and the second (dashed line) Brillouin zone of graphene. Red solid lines represent cuts in the band structure associated to the width of the considered AGNR; (e) Tight-Binding band structure of graphene; (f-h) Band structure of the 5- (f), the 4- (g) and the 3-AGNR (h) deduced from cuts in the graphene dispersion.

Supplementary Notes

Supplementary Note 1: Growth of 1,4-dibromo-benzene (dBB) on Cu(110) as function of coverage.

As expected for Ullmann coupling, as well as in agreement with previous experimental[1-5] and theoretical[4] studies, the evaporation of the dBB molecules on Cu(110) held at room temperature leads to the appearance of an organometallic (OM) phase, where phenyl rings are bonded to copper atoms. However, our investigation shows that at higher molecular flux the structure of the OM phase depends critically on the number of molecules deposed on the substrate. At low coverage (<0.8 ML, estimated using the evaporation time required to reach saturation), the superstructure we observe is identical to the one described previously by M. Di Giovannantonio et al. (figure S1a)[2]. At higher coverage (>0.8 ML), a new phase emerges (Saturated-OM), which coexists with the previous one (Unsaturated-OM), as shown in the figure S1b. As the coverage increases, the ratio between the saturated and unsaturated phases also increases, until only the first one remains at1 ML (figure S1c). At this point, it becomes impossible to increase again the coverage, revealing a self-limited process. STM, STS and XPS measurements performed on the saturated phase confirm its organometallic-nature. A detailed investigation of this novel reconstruction and the phase transition mechanism will be provided in a future publication.

Annealing of the two OM phases leads in both cases to the formation of the one-dimensional poly-para-phenylene (PPP), but aligned along different crystallographic directions. As shown in figure S1d, annealing up to 475K of the Unsaturated-OM leads to the production of unidirectional polymers chains oriented along <1,-1,0> [2]. The details of this structure are discussed in note 2. The annealing of a sample containing the two different OM phases leads to the coexistence of the <1,-1,0> “aligned” PPP and the “transverse” polymer oriented in <1,-1,2> and <1,-1,-2> directions (figure S1e). Again, as the coverage increases, the ratio between the transverse and the aligned chains increases and only the “transverse” PPP is observed for 1 ML.

Since the four phases (2 OMs and 2 polymers) are characterized by different unit cells, LEED measurements can easily be used to identify the different reconstructions, as was done at the CASSIOPEE beamline at SOLEIL. Figure S2 shows LEED patterns recorded at 72 eV for the four different

superstructures, and also for intermediate coverage where a coexistence of both phases is visible (figure S2b,e). Red and blue arrows indicate characteristic diffraction spots of the unsaturated and saturated superstructures, respectively. We note that the saturated OM is characterized by the appearance of three prominent spots between those of the substrate (green circles) along the <0,0,1> azimuth (figure S2b,c). This pattern can easily be distinguished from the <1,-1,0>-aligned polymer's, since only two additional spots appear in the same direction (figure S2d). Finally, the transverse polymer, obtained annealing a saturated surface, is characterized by spots forming lines rotated by ± 35º from the <1,-1,0> direction (figure S2e,f).

Supplementary Note 2: Structure and commensurability of the polymers along <1-10> and <1-12>.

As discussed above, annealing an unsaturated DBB layer on Cu(110) surface leads to the formation of the PPP aligned along the <1,-1,0> direction. Figure 3a contains a high resolution STM image of this structure. Phenyl rings (white hexagons) are covalently bonded, forming the polymer which can also be interpreted as the smallest hydrogen passivated graphene nanoribbon in the armchair conformation (3-AGNR, see note 7). STM data and DFT calculations both suggest the inter-phenyl distance is close to 4.4 Å. NEXAFS measurements show that the polymer is completely planar and parallel to the surface.[2] The PPP chains are separated in the perpendicular direction by bromine atoms (green balls), covalently bonded to the surface on the short bridge (SB) sites, forming a linear and commensurate arrangement with the substrate, characterized by a Br-Br distance of 5.1 Å (twice the substrate periodicity along <1,-1,0>. Indeed, as shown in figure S3b, in the <1,-1,0> direction, the Cu lattice parameter is 2.55 Å, which implies incommensurability between the polymer and the surface.

However, when the surface is saturated by dBB, annealing induces the formation of PPP chains aligned along <1,-1,2> and <1,-1,-2> (figure S3c). The reconstruction is qualitatively similar to the one observed at low coverage: phenyl rings are bonded in the armchair geometry and chains are separated by lines of atomic bromine. Close inspection of STM pictures reveals that the inter-phenyl and Br-Br distance are exactly the same, measured at 4.4±0.2 Å. A DFT relaxation of the system has shown that Br

atoms are again chemisorbed in SB sites in a commensurate geometry (figure S3d). In this particular structure the surface lattice parameter in the <1,-1,2> is 4.43 Å., leadings to the commensurability between the polymer and the surface.

Supplementary Note 3: Long range order at the saturation coverage and STS measurements.

A large scale STM image of the reconstruction obtained after polymerization of 1ML dBB/Cu(110) is shown in figure S4a. The polymer is aligned in domains along both <1,-1,2> and <1,-1,-2> directions (yellow arrows), and these domains are present in equal proportions. As the polymer unit cell contains only one bromine atom per phenyl ring, the straight lines running along <1,-1,0> directions are composed of bromine atoms. The size distribution of the PPP chains has been studied as function of annealing temperature on long-range STM images. The results are presented in the inset of figure S4a. Annealing the surface just above the temperature transition, at 475K for five minutes leads to a mean length of 15 phenyl rings (≈7 nm). Increasing up to 525K, this length is increased to almost 20 phenyl rings (≈9 nm). The size distribution becomes asymmetric, increasing the number of long oligomers beyond 15 nm. Longer annealing or higher temperature have not been tested. Nevertheless, the electronic properties of a 20 phenyl rings chain are quite similar to those of an infinitely long polymer (see figure 2a and 3b in the main text). Figure S4b is a high resolution STM image of the area containing the polymers of N phenyl rings with N = 6, 10, 13, 17 and 22, used to record STS measurements presented in the figure 2 of the main text. In order to characterize the evolution of LUMO states as function of the chain's length, several conductance spectra have been acquired in different positions for each chain, close to L/2 (green point) and L/3(blue point). Results are displayed in figure S4c. Experimental spectra (colored points) are fitted (solid lines) removing first a polynomial background and using several Gaussian contributions. According to the classical model of standing waves in a 1D quantum well, the spectrum recorded at L/2 allow us to measure the position of the LUMO, LUMO+2 and LUMO+4. On the one recorded at L/3 we can follow the evolution of the LUMO, LUMO+1 and LUMO+3. We can also notice that, in average, the lifetime of electrons is highly reduced while the chain length is increased.

Supplementary Note 4: Molecular origin of the dispersion.

The comparison of the ARPES intensity maps recorded in the <1,-1,2> direction (parallel to the chains) on the clean surface (figure S5a) and the polymerized 1ML-dBB/Cu(110) (figure S5b) has allowed us to attribute the observed dispersion to the molecular layer, indicated by yellow arrows, which cross the d states of the substrate. In addition, measurements as function of the light polarization provided us information on the symmetry of these states. Indeed, NEXAFS measurements performed on the <1,-1,0> aligned polymer have shown that this one is planar, with all the phenyl rings parallel to the surface.[2] Our STM high resolution images in conjunction with our DFT calculations lead to the conclusion that the <1,-1,2> and <1,-1,-2> aligned polymers are also flat. In that case, the complete extinction of the molecular signal observed using s-polarization (in surface plane polarization, perpendicular to the π orbitals axis) is in agreement with the π symmetry of these electronic states (figure S5c). This result, as well as the band structure and spectral intensity theoretically predicted by the tight-binding model, allow us to conclude on the π nature of the observed dispersive band.

Supplementary Note 5: ARPES intensity calculation in the Hückel model.

The Hückel method permits calculation of the π orbitals of small 2D conjugated hydrocarbon molecules such as finite PPP chains. In first approximation, we consider only the Coulomb and hopping integrals between p_z orbitals of two neighboring atoms. Diagonalizing the Hamiltonian as-defined, we calculate all the π orbitals of PPP chains as function of the length, i.e. the number of phenyl rings. Figure S6a shows some of orbitals calculated for the sexiphenyl (N=6). We note that the shape of the HOMO is in good agreement with the one obtained by DFT.[6] Then, according to Fermi's golden rule and the method introduced by P. Puschnig et al.[6], we build the corresponding Constant Energy ARPES Map (CEAM) for each orbital using a simple Fourier transformation, as shown in figure S6b. Since energy levels of each molecular orbital are known, we can deduce from all the CEAMs the ARPES intensity map as function of energy and wave vector (figure S6c). The broadening in energy is empirically determined

in order to reproduce experimental measurements. The result obtained for the sexiphenyl is found to be in very good agreement with experimental data obtained by G. Koller et al.[7]

As mentioned above, these calculations can be repeated as function of the PPP length. Comparing the E vs $k_{//}$ dispersion of biphenyl (N=2) and sexiphenyl (N=6), we observe for the latter the emergence of a pseudo-dispersion, associated with the 1D infinite polymer states confined in the parallel direction by the finite length of the molecule (figure S7). When the length is increased, the effect of the confinement is reduced. For a chain containing 20 phenyl rings, the calculated dispersion is virtually indistinguishable from the one expected for the infinite polymer.

Supplementary Note 6: Hückel estimation of the perpendicular coupling.

As discussed in the main text, the perpendicular modulation of polymer contributions observed on the CEAMs is interpreted as an inter-chain coupling possibly mediated by the substrate and/or bromine atoms separating polymers (see Figures 3f in the main text and figure S8a). Using the Hückel model, we can estimate the value of an effective hopping integral β' between the electronic states of these chains characterizing the substrate-mediated interaction. First, the Hamiltonian is defined and diagonalized for an isolated chain containing 20 phenyl rings, using β=-3.5 eV as hopping integral between the first neighboring carbons atoms. Then, β' is introduced as a coupling constant between the deduced molecular orbitals of three chains separated by a perpendicular distance of 10.4 Å, corresponding to our STM measurements (figure S8b,c). The Fourier transforms of the coupled orbitals, broadened in energy by a 200 meV Lorentzian contribution, allow us to reconstruct the ARPES theoretical dispersion. Subsequently a constant energy cut, taken at -1.8 eV, reveals the 1D contributions which are modulated in the perpendicular direction with a periodicity of 2π/b (b=10.4 Å) as observed experimentally (figure S8a). The amplitude of this modulation is directly related to the value of β' and the best agreement with experimental measurements is found using β' = -0.15 ± 0.05 eV.

Supplementary Note 7: Why is PPP a graphene nanoribbon?

From the geometrical point of view, one can define graphene nanoribbons by cutting a graphene layer in a selected orientation, (ΓM) in the case of armchair graphene nanorribons (AGNRs). From an electronic point of view, a GNR is any one-dimensional system for which electronic properties can be deduced considering only those of graphene, taking into account the confinement of electrons in the perpendicular direction. This definition means that, in the case of n-AGNR, the one-dimensional band structure can be built by taking cuts into the two-dimensional graphene one at constant perpendicular wave vector $k^{j}_{\perp} = 2.\pi.j / (a.(n+1))$ with $1 \leq j \leq n$ and a the lattice parameter of the graphene. The as-deduced band structures for the 5-, the 4- and the 3-AGNR using the tight-binding model are shown in figure S9. We notice that the one of the 3-AGNR matches with the band structure calculated for the PPP (see also Figure 4 in the main text). Thus, the comparison of PPP to an AGNR is acceptable from this point of view.

Supplementary Note 8: Gas phase DFT calculations

Gas phase DFT calculations have been carried out using Gaussian 09 at the B3LYP/631g(d) level.[8] This gives a band gap of 3.05 eV for PPP in agreement with the expected value.[9]

Supplementary References :

1. Lipton-Duffin J., Ivasenko O., Perepichka D., and Rosei F., Synthesis of polyphenylene molecular wires by surface-confined polymerization, *Small* **5**, 592 (2009).
2. Di Giovannantonio et al., Insight into organometallic intermediate and its evolution to covalent bonding in surface-confined Ullmann polymerization, *ACS Nano* **7**, 8190-8198 (2014).
3. Di Giovannantonio et al., Reply to "Comment on 'Insight into Organometallic Intermediate and Its Evolution to Covalent Bonding in Surface-Confined Ullmann Polymerization", *ACS Nano* **8** (3), 1969 (2014).
4. Wang W. et al., Single-molecule resolution of an organometallic intermediate in a surface-supported ullmann coupling reaction, *J. Am. Chem. Soc.* **133**, 13264–13267 (2011).
5. Bjork J., Hanke F., and Stafstrom S., Mechanisms of halogen-based covalent self-assembly on metal surfaces, *J. Am. Chem. Soc.* **135**, 5768–5775 (2013).
6. Puschnig P. et al., Complete determination of molecular orbitals by measurement of phase symmetry and electron density, *Science* **326**, 702 (2009).
7. Koller G. et al., Intra- and Intermolecular Band Dispersion in an Organic Crystal, *Science* **317**, 351 (2007).
8. M. J. Frisch, G. W. T., H. B. Schlegel, G. E. Scuseria, M. A. Robb, J. R. Cheeseman, J. A. Montgomery, Jr., T. Vreven, K. N. Kudin, J. C. Burant, J. M. Millam, S. S. Iyengar, J. Tomasi, V. Barone, B. Mennucci, M. Cossi, G. Scalmani, N. Rega, G. A. Petersson, H. Nakatsuji, M. Hada, M. Ehara, K. Toyota, R. Fukuda, J. Hasegawa, M. Ishida, T. Nakajima, Y. Honda, O. Kitao, H. Nakai, M. Klene, X. Li, J. E. Knox, H. P. Hratchian, J. B. Cross, V. Bakken, C. Adamo, J. Jaramillo, R. Gomperts,

R. E. Stratmann, O. Yazyev, A. J. Austin, R. Cammi, C. Pomelli, J. W. Ochterski, P. Y. Ayala, K. Morokuma, G. A. Voth, P. Salvador, J. J. Dannenberg, V. G. Zakrzewski, S. Dapprich, A. D. Daniels, M. C. Strain, O. Farkas, D. K. Malick, A. D. Rabuck, K. Raghavachari, J. B. Foresman, J. V. Ortiz, Q. Cui, A. G. Baboul, S. Clifford, J. Cioslowski, B. B. Stefanov, G. Liu, A. Liashenko, P. Piskorz, I. Komaromi, R. L. Martin, D. J. Fox, T. Keith, M. A. Al-Laham, C. Y. Peng, A. Nanayakkara, M. Challacombe, P. M. W. Gill, B. Johnson, W. Chen, M. W. Wong, C. Gonzalez, and J. A. Pople, Gaussian, Inc., Wallingford, CT, 03, Revision D.01 ed., 2004.

9. Giro R., Caldas M. J. & Galvão D. S., Band gap engineering for poly(p-phenylene) and poly(p-phenylene vinylene) copolymers using the tight-binding approach, *Int. J. Quantum Chem.* **103**, 588 (2005).
